# Simvastatin Improves the Jaw Bone Microstructural Defect Induced by High Cholesterol Diet in Rats by Regulating Autophagic Flux

**DOI:** 10.1155/2018/4147932

**Published:** 2018-06-27

**Authors:** Jianhua Zhou, Xiaoli Gao, Shengyun Huang, Li Ma, Yanjun Cui, Hengkun Wang, Jianzhong Qiu, Lili Wang, Quanjiang Dong, Zhenggang Chen, Xuxia Wang, Dongsheng Zhang

**Affiliations:** ^1^Department of Oral and Maxillofacial Surgery, Shandong Provincial Hospital, Affiliated to Shandong University, Jinan, Shandong 250012, China; ^2^Key Laboratory of Shandong Provincial Hospital, Jinan, Shandong 250012, China; ^3^Department of Oral and Maxillofacial Surgery, School of Stomatology, Shandong University, Jinan, Shandong 250012, China; ^4^Department of Stomatology, Qingdao Municipal Hospital, Qingdao, Shandong 266071, China; ^5^Central Laboratories, Qingdao Municipal Hospital, Qingdao, Shandong 266071, China

## Abstract

**Objective:**

The objective of this study is to evaluate the effect of simvastatin on the jaw bone microstructural defect and autophagy in rats with high cholesterol diet (HCD).

**Methods:**

Male Sprague-Dawley rats were fed a standard rodent chow (NC group) or a high cholesterol diet for 32 weeks and the HCD-fed rats were treated with vehicle (HC group) or simvastatin (5 mg/kg orally daily for 8 weeks, HC + SIM group, and *n* = 10/group). The static histomorphometric changes in the jaw bone tissues in individual rats were evaluated. The relative levels of OPG, RANKL, NF-*κ*B, LC3, and p62 in the jaw bone tissues were determined by quantitative RT-PCR and/or immunohistochemistry.

**Results:**

Compared with the NC group, the HC groups had lower trabecular bone volume, trabecular thickness and trabecular number, and increased ratios of RANKL/OPG in the jaw bone, accompanied by enhanced NF-*κ*B activation and autophagy. Simvastatin treatment inhabited these changes, including the decreased levels of serum proinflammatory cytokines and increased autophagy.

**Conclusion:**

Simvastatin treatment could inhibit the hyperlipidemia-induced jaw bone microstructural defect in rats by increasing autophagic flux.

## 1. Introduction

High fat or cholesterol diet can cause hyperlipidemia, which is associated with oxidative stress and inflammation, leading to osteoporosis, a high prevalent metabolic disorder in developed and developing countries [1]. Previous studies have shown that hyperlipidemia is associated inversely with the levels of bone mineral density (BMD) in the jaw [2–5]. Low BMD will affect the osseointegration, stability, and function of an implant [6]. However, some studies have revealed that high fat diet containing diacylglycerol mitigates high fat diet-induced bone metabolic dysfunction in mice and promotes peak bone mass in young rats [7, 8]. Therefore, the precise effect of high fat diet, particularly for high cholesterol diet (HCD), on the bone metabolism in the jaw remains controversial.

Autophagy is an intracellular process of degradation of cytoplasmic components in lysosomes to maintain cellular homeostasis. During the process of autophagy, many stimuli can induce the formation of autophagosomes, in which the microtubule-associated protein 1A light chain 3 (LC3-I) on the autophagosomal inner membrane converts to LC3-II by conjugating to phosphatidylethanolamine. The LC3-II can recruit p62/SQSTM1 promotes the degradation of cargo proteins [9]. Recent studies have reported that autophagy is crucial for the function and survival of osteoclasts, osteoblasts, and osteocytes, as well as chondrocytes [10, 11]. However, the role of autophagy in regulating the hyperlipidemia-induced jaw bone abnormality has not been clarified.

Statins are common drugs for the treatment of hyperlipidemia and can modulate lipid and bone metabolism, improving osteoporosis [12–14]. Previous studies have shown that treatment with statin drug not only minimizes the risk of hyperlipidemia-related bone fracture but also increases osteoblastic activity [15, 16]. Actually, treatment with simvastatin can increase the osteogenesis and inhibit bone reabsorption in the parietal bone of rabbits [17]. It is notable that the jaw bone originates from the neural crest ectoblast mesenchyme with a distinct intramembranous ossification and has a better osteogenic differentiation ability [18, 19]. However, there is no information on whether and how treatment with simvastatin can modulate the hyperlipidemia-induced jaw bone microstructural defect.

In this study, we tested the hypothesis that treatment with simvastatin could mitigate hyperlipidemia-induced jaw bone microstructural defect by upregulating autophagy flux in rats.

## 2. Materials and Methods

### 2.1. Animals and Experimental Design

Male Sprague-Dawley rats at 6 weeks of age and weighing 170-190 g were obtained from Beijing Vital River Laboratory Animal Technology and housed in a specific pathogen-free facility at 22-25°C with a 12-hour light/dark cycle and free access to standard rodent chow and water. After one week of acclimatization, the rats were randomized into (1) normal control diet (the NC group, *n* = 10) with 100% standard rodent chow; or (2) high cholesterol diet (HCD, *n* = 20) with 2% cholesterol + rodent chow (Table 1, Beijing Vital River Laboratory Animal Technology) for 24 weeks. The NC group of rats continually received standard rodent chow. The HCD-fed rats were continually fed with HCD, randomized, and treated orally with vehicle as the HC group or with 5 mg/kg simvastatin by gavage daily for eight weeks as the HC + SIM group. During the experiment period, body weights of individual rats were measured using an automatic electronic balance (Shanghai Yousheng Weighing Apparatus, Shanghai, China) weekly. The experimental protocol was approved by the Animal Ethics Committee of Shandong Provincial Hospital.

### 2.2. Serum Analysis

At thirty-two weeks after HCD feeding, the rats were food-fasted overnight and their peripheral blood samples were collected, followed by sacrifice. After coagulation of the collected blood samples, the blood samples were centrifuged for preparing serum samples. The levels of serum triglycerides (TG), total cholesterol (TC), high-density lipoprotein (HDL), and low-density lipoprotein (LDL) were measured using enzymatic methods in an AutoBiochemical Analyzer (AU5400; Olympus Corporation, Tokyo, Japan).

### 2.3. Bone Histology

After being sacrificed, the jaw bone tissue samples from individual rats were dissected out and fixed in 4% paraformaldehyde for 48 hours. After fixation, the jaw bone and tooth samples were decalcified in 10% EDTA solution (pH 7.2) for 2 weeks at room temperature. The decalcifying solution was changed with fresh one every 5 day. The samples were paraffin-embedded, and the longitudinal tissue sections (5 *μ*m) were stained with hematoxylin and eosin (H&E). Three to five areas selected randomly from each sample were photoimaged under an Olympus microscope (BX51TRF; Tokyo, Japan) using the Image Pro Plus software, version 6.0 (Media Cybernetics, Rockville, MD, USA). The trabecular bone volume relative to the tissue volume (BV/TV, %), trabecular number (Tb.N, mm^−1^), trabecular thickness (Tb.Th, *μ*m), and trabecular separation (Tb.Sp, *μ*m) in each image were analyzed in a blinded manner.

### 2.4. Immunohistochemistry

The bone tissue sections were deparaffinized using xylene and rehydrated by serial concentrations of ethanol. After antigen retrieval using 0.125% pancreatin at 37°C for 30 min, the sections were treated with 3% H_2_O_2_ in methanol to inactivate endogenous peroxidase and were incubated with anti-NF-*κ*B (1 : 200), anti-LC3 (1 : 100), and anti-p62 (1 : 200, Abcam, Cambridge, UK) at 4 °C overnight. After being washed, the sections were incubated; biotinylated secondary antibodies and the bound antibodies were detected with peroxidase-labeled streptavidin at 37 °C for 30 min. The stained signals in the sections were visualized using Diaminobenzidine (Zsgb-bio, Beijing, China) and the sections were counterstained with haematoxylin. The vehicle PBS was used as the negative control in every step.

The intensity of the IHC staining was evaluated semiquantitatively using Image Pro Plus software, as described previously [20, 21]. Six visual fields selected randomly from each section were photoimaged and the mean values (IOD sum/area sum) of the images (magnification, 400×) were designated as representative staining intensity of LC3-II, p62, and NF-*κ*B to determine the relative expression levels. The images were analyzed by two specialized pathologists in a blinded manner.

### 2.5. Reverse Transcription and Quantitative PCR

The dissected jaw bone tissues were frozen in liquid nitrogen and powdered. Total RNA was extracted from each jaw bone sample using Trizol reagent (Ambion), according to the manufacturer's instruction. The RNA samples were reversely transcribed into cDNA using the PrimeScript™ RT reagent kit with gDNA Eraser (TakaRa, Japan), according to the manufacturer's instruction. The relative levels of target gene mRNA transcripts to the control GAPDH were analyzed by quantitative RT-PCR using the FastStart DNA Master SYBR Green I kit (TakaRa, Japan) and specific primers in a LightCycler system 480 (Roche). The sequences of primers for OPG, RANKL, NF-*κ*B, LC3, p62, and GAPDH are shown in Table 2. The PCR reaction consisted of 25 *μ*l of 1 × SYBR Premix Ex TaqII, 0.2 *μ*mol/l each of primers, and 20 ng cDNA template. The amplifications were performed in duplicate at 95°C for 30 s and subjected to 40 cycles of 95° C for 5 s and 60°C for 30 s. The relative levels of each target gene mRNA transcripts to the GAPDH were analyzed using 2^-△△Ct^ method [22] and expressed as relative value (fold change) to the levels in the NC group.

### 2.6. Statistical Analysis

Data are expressed as mean ± standard deviation (SD). The difference among groups was assessed by one-way analysis of variance (ANOVA) and post hoc Fisher's Least Significant Difference (LSD) test using the SPSS, version 17.0 (SPSS, Chicago, IL, USA). A *P* value of < 0.05 was considered statistically significant.

## 3. Results

### 3.1. Treatment with Simvastatin Significantly Improves Hyperlipidemia in Rats Fed with HCD

**Figure 1:**
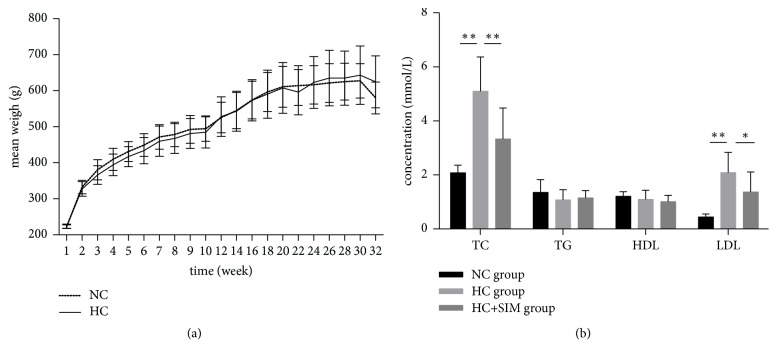
**The effect of a high cholesterol diet and simvastatin treatment on rat body weight and serum lipid profile.** (a) The body weights of the rats were monitored weekly. (b) Serum triglycerides (TG), total cholesterol (TC), high-density lipoprotein (HDL), and low-density lipoprotein (LDL) levels in rats assayed in the 32th week. Results are expressed as mean ± SD of each group. NC: normal control diet; HC: high cholesterol diet; HC+SIM: high cholesterol diet + simvastatin; ^*∗*^*P* < 0.05; ^*∗∗*^*P* < 0.01.

To determine the effect of simvastatin on HCD-induced hyperlipidemia, SD rats were fed with HCD for 24 weeks, randomized, and treated orally with vehicle or simvastatin daily for 8 weeks. A group of rats received vehicle PBS as the NC controls. Their body weights were measured weekly in Figure 1. All groups of rats increased their body weights gradually and there was no statistical significance in the body weights among these groups of rats at any specific time points measured (*P *> 0.05, Figure 1(a)).

Analysis of serum lipids indicated that there was no significant difference in the levels of serum TG and HDL among these groups of rats. In comparison with that in the NC group, significantly higher levels of TC and LDL were detected in the HC group (*P* < 0.001). However, the levels of serum TC and LDL in the HC + SIM group were significantly lower than that in the HC group (*P* < 0.01) but remained significantly higher than that in the NC group (*P* < 0.01, Figure 1(b)). Hence, treatment with simvastatin did not change the body weights but significantly mitigated the HCD-induced hyperlipidemia in rats.

### 3.2. Treatment with Simvastatin Improves the Hyperlipidemia-Induced the Jaw Bone Microstructural Defect in Rats

Hyperlipidemia can induce oxidative stress, leading to the bone microstructural defect. Next, we examined the effect of simvastatin on the jaw bone microstructure in the HCD-fed rats by histology. The static histomorphometry revealed that the HCD-fed rats displayed significantly lower trabecular bone volume (BV/TV), trabecular thickness (Tb.Th), and trabecular number (Tb.N), with the trabecular separation (Tb.Sp) higher than the NC rats (*P* < 0.05, Table 3). In contrast, the BV/TV, Tb.Th, and Tb.N values in the HC + SIM group of rats were significantly higher than HC group (*P* < 0.05), while the Tb.Sp values were significantly lower than that of the HC group of rats (*P* < 0.05). More importantly, there was no significant difference in the values of these measures between the HC + SIM and NC groups of rats.

**Figure 2:**
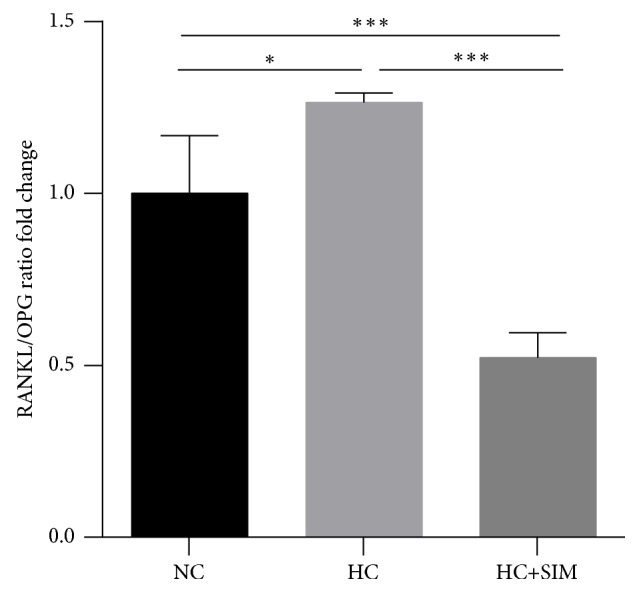
**Effects of simvastatin on the jaw bone tissue in HC rats.** RANKL/OPG ratios were assessed by qPCR. Results are expressed as mean ± SD of each group. NC: normal control diet; HC: high cholesterol diet; HC + SIM: high cholesterol diet + simvastatin; ^*∗*^*P* < 0.05; ^*∗∗∗*^*P* < 0.001.

Next, the relative levels of RANKL and OPG mRNA transcripts in the jaw bone tissues of individual rats were determined by quantitative RT-PCR and the ratios of RANKL to OPG mRNA transcripts in individual groups of rats were calculated (Figure 2). In comparison with the NC group, significantly higher ratios of RANKL to OPG mRNA transcripts were detected in the jaw bone tissues of the HC groups of rats (*P* < 0.05). Treatment with simvastatin significantly decreased the ratios of RANKL to OPG mRNA transcripts in the HC + SIM group (*P* < 0.001 vs. the NC or HC, Figure 2). Collectively, treatment with simvastatin significantly mitigated the hyperlipidemia-induced jaw bone microstructural defect in rats.

### 3.3. Treatment with Simvastatin Inhibits Hyperlipidemia-Related Inflammation in the Jaw Bone of HCD-Fed Rats

**Figure 3:**
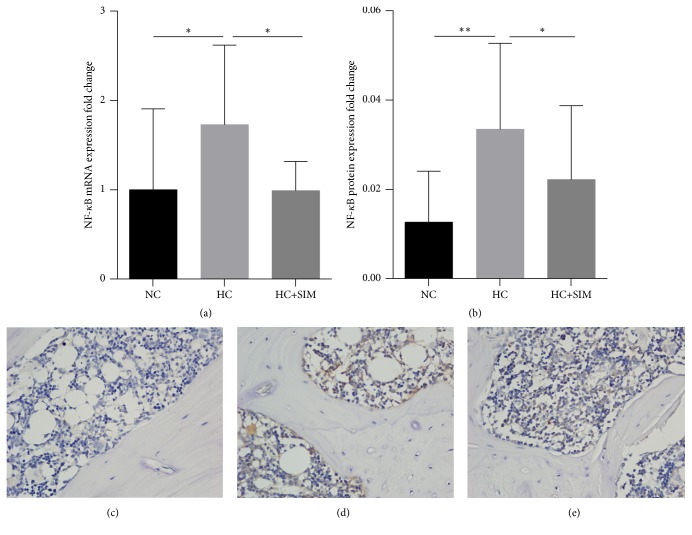
**Effects of simvastatin on the NF-*κ*B signaling in the jaw bone of HC rats.** HCD feeding increased the levels of NF-*κ*B signaling in the jaw bone tissues. Simvastatin reduced the one. (a) NF-*κ*B gene expression. (b) NF-*κ*B protein expression in NC (c), HC (d), and HC+SIM (e) (magnification, 400). NC: normal control diet; HC: high cholesterol diet; HC+SIM: high cholesterol diet + simvastatin; ^*∗*^*P* < 0.05; ^*∗∗*^*P* < 0.01; ^*∗∗∗*^*P* < 0.001.

Hyperlipidemia can induce systemic inflammation and increase the NF-*κ*B signaling and TNF-*α* and IL-1*β* expression in animals. To understand the action of simvastatin, the relative levels of NF-*κ*B mRNA transcripts in the jaw bone tissues of individual rats were determined by quantitative RT-PCR. In comparison with that in the NC group, significantly increased levels of NF-*κ*B mRNA transcripts were detected in the HC group (*P* < 0.05, Figure 3(a)). Treatment with simvastatin significantly reduced the levels of NF-*κ*B mRNA transcripts by 39.34% compared to those in the HC group (*P* < 0.05). Further immunohistochemistry revealed that the NF-*κ*B was expressed in osteoblasts, inflammatory mononuclear infiltrates, bone-lining cells, and osteoclasts. The mean density of anti-NF-*κ*B immunoreactivity in the bone tissues of the HC group was significantly higher than in the NC (*P* < 0.01) and HC + SIM groups (*P* < 0.05, Figures 3(b)–3(e)). Therefore, treatment with simvastatin mitigated the hyperlipidemia-induced inflammation in the jaw bone tissues of rats.

### 3.4. Treatment with Simvastatin Increases the Hyperlipidemia-Induced Autophagic Flux in the Jaw Bone Tissues of Rats

**Figure 4:**
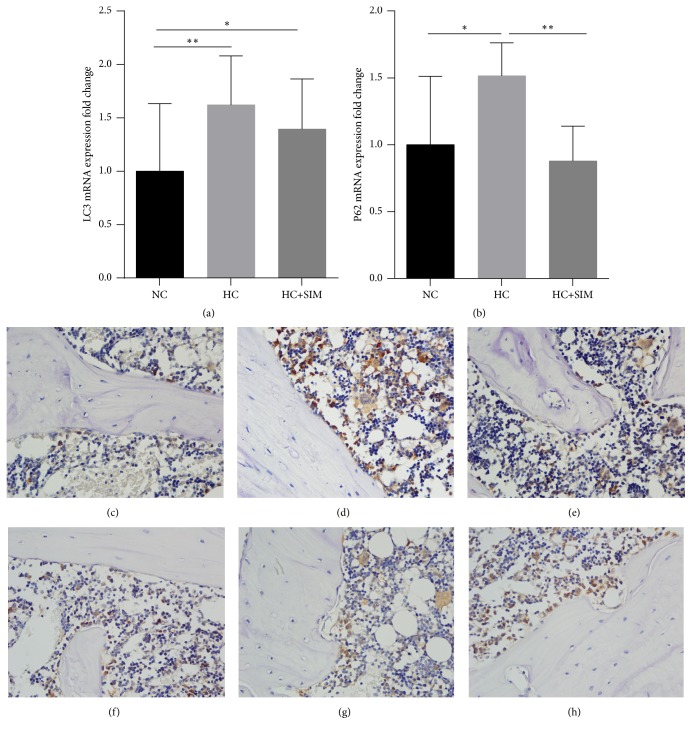
**Effects of simvastatin on the autophagic regulation in HC rats.** The levels of LC3 (a) and p62 (b) expression were assessed by qPCR. LC3 positive cells were observed in NC (c), HC (d), and HC + SIM (e), and p62 positive cells were observed in NC (f), HC (g), and HC + SIM (h) by IHC. Results are expressed as mean ± SD of each group. NC: normal control diet; HC: high cholesterol diet; HC + SIM: high cholesterol diet + simvastatin; IHC: Immunohistochemistry; ^*∗*^*P* < 0.05; ^*∗∗*^*P* < 0.01; ^*∗∗∗*^*P* < 0.001.

Hypercholesterolemia can inhibit autophagy in the heart of rats [23]. Finally, we examined the effect of simvastatin on autophagic flux in the jaw bone tissues of HCD-fed rats by quantitative RT-PCR and immunohistochemistry. In comparison with that in the NC group, significantly higher levels of LC3 and p62 mRNA transcripts were detected in the jaw bone tissues of the HC group of rats (*P* < 0.01,* P* < 0.05, respectively, Figures 4(a) and 4(b)). However, the relative levels of p62 mRNA transcripts in the HC + SIM group were significantly lower than that in the HC group (*P* < 0.01). Immunohistochemistry revealed similar patterns of the levels of LC3 and p62 protein expression in the jaw bone tissues of the different groups of rats (Figures 4(c)–4(h), Table 4). Thus, treatment with simvastatin increased the hyperlipidemia-induced autophagic flux in the jaw bone tissues of HCD-fed rats.

## 4. Discussion

The present study employed a HCD-fed rat model to test the effect of simvastatin treatment on the jaw bone microstructural defect. We found that treatment with simvastatin not only improved the HCD-induced hyperlipidemia but also mitigated the HCD-induced jaw bone microstructural defects by reducing inflammation and increasing autophagic flux in the jaw bone of rats. To the best of our knowledge, this was the first study to show that autophagy regulated the jaw bone metabolism in rats fed with HCD.

Feeding with HCD or HFD can induce hyperlipidemia in animals. We found that rats fed with HCD for 32 weeks had significantly higher levels of serum TC and LDL to develop hyperlipidemia, consistent with a previous observation [24]. Treatment with simvastatin significantly decreased the levels of serum TC and LDL in rats. High levels of serum LDL are risk for bone tissue alterations [25–27]. Indeed, hyperlipidemia increases the osteoclast numbers and reduces the alveolar bone density in rats [28]. We found that HCD-fed rats developed significantly lower trabecular bone volume, trabecular thickness, and trabecular number but higher trabecular separation, demonstrating the jaw bone microstructural defects. In contrast, treatment with simvastatin improved the hyperlipidemia-induced jaw bone microstructural defects in rats. Hence, long-term HCD may have an adverse effect on dental implant and denture restoration. It is well known that old people, particularly for those with age of >55 years, usually have deficient in osteogenesis but increased levels of lipogenesis, leading to a significant decrease in bone density and a potential failure of dental implants [29, 30]. Conceivably, treatment with simvastatin to reduce hyperlipidemia may benefit patients with dental implants, particularly those old people, by preventing/inhibiting the hyperlipidemia-induced bone microstructural defects.

It is well known that the balance of RANKL and OPG is crucial for regulating osteoclastogenesis and osteoblastogenesis, contributing to the bone metabolism and remodeling [31]. High-level RANKL produced by osteoblasts can bind to its receptor of RANK and stimulate osteoclast differentiation by activating the NF-*κ*B signaling to induce c-Fos expression and NFATc1-triggered osteoclastogenic gene transcription, leading to osteoclastogenesis and bone resorption [31–33]. OPG, a decoy receptor of RANKL, can bind to RANKL and prevent the RNAKL/RANK-mediated NF-*κ*B activation to inhibit osteoclast differentiation but promote osteoblast differentiation in the bone. While hyperlipidemia can inhibit the proliferation and differentiation of osteoblasts and statins, such as simvastatin, it can stimulate osteoblast differentiation [34–36]. Indeed, treatment with simvastatin can promote the bone formation and prevent bone loss in animals with inducible periodontitis by inhibiting inflammation [37]. In addition, simvastatin may stimulate OPG production [38–40]. In this study, we found that HCD-related hyperlipidemia also enhanced the NF-*κ*B expression in the jaw bone of rats, which was demolished by simvastatin treatment. Therefore, simvastatin treatment suppressed osteoclast differentiation and promoted osteoblast differentiation by down-regulating the RANKL/OPG ratio and NF-*κ*B activation in rats.

A previous study has shown that chronic HFD feeding can decrease the levels of autophagy in mouse hypothalamus [41]. Inhibition of autophagy can reduce the expression of master regulators of lipid metabolism and enhance the NF-kB signaling and inflammatory response. The sterol regulatory element binding protein (SREBP-2), a transcription factor that regulates cholesterol metabolism, can activate autophagy [42]. A recent study indicates that genetic or pharmacological inhibition of autophagy suppresses mesenchymal stem cell differentiation into osteoblasts [43]. Similarly, autophagy appears to be crucial for ruffled border formation, secretion, and bone resorption of osteoclasts in vitro and in vivo [44, 45]. Ubiquitin binding proteins, such as p62, can regulate these ubiquitin-mediated processes and enhance the RANK-NF-*κ*B signaling, which promotes osteoclastogenesis and osteoclast formation [46]. In this study, we found that HCD-fed increased the levels of LC3-II and p62 expression in the jaw bone of rats, consistent with other studies [47–51]. Sabe et al. [51] thought that the high expression of LC3-II only could suggest either an increase in autophagy or a decrease in autophagy, because these proteins also accumulate in the environment of decreased autophagy. Furthermore, detection of the autophagy flux marker p62 can make more clear understanding of the alterations. The higher levels of p62 may stem from diminished autophagy due to the abnormality of autolysosomal degradation. More importantly, treatment with simvastatin prevented these changes and increased autophagy flux in the jaw bone of rats. These novel findings suggest that autophagy may regulate the jaw bone metabolism in HCD-fed rats.

## 5. Conclusion

In conclusion, the present study revealed that HCD feeding induced hyperlipidemia and decreased trabecular volume by increasing the RANKL/OPG ratios and activating the NF-*κ*B signaling, which modulated autophagy in the jaw bone of rats. Simvastatin treatment improved the hyperlipidemia-induced jaw bone microstructural defects by increasing autophagy flux. Potentially, our findings may provide a significant contribution to further experimental and clinical studies by directly comparing the effects of simvastatin on the jaw bone with other bone tissues.

## Figures and Tables

**Table 1:** The composition of the high cholesterol diets.

	Normal control diet	High cholesterol diet
Protein (g/100g)	20	17
Carbohydrate (g/100g)	58	49
Fat (g/100g)	6	21
Selenium (g/100g)	1.4×10^−5^	1.6×10^−5^
Cholesterol (g/100g)	0	2
Fatty acids (g/100g)		
C14:0	0.02	0.02
C16:0	0.97	0.97
C16:1	0.02	0.02
C18:0	0.21	0.21
C18:1	1.23	1.23
C18:2	2.57	2.57
C18:3	0.17	0.17
Total saturated	1.11	1.11
Total monounsaturated	1.22	1.22
Total polyunsaturated	2.93	2.93
Total kcal/g	3.4	3.4

**Table 2:** The sequence of primers used in the polymerase chain reaction (PCR) study.

Primer	5′-3′ sequences
GAPDH	F:CCCTCTCTCTGCTCACTCTGCT R: CTTACTGCCCTCCTGCTTGG
OPG	F: TGTGGAATAGATGTCACCCTGTGC R: CACAGAGGTCAATGTCTTGGATGATC
RANKL	F: GCTTCTCAGGAGTTCCAGCTATGAT R: CGTTGCTTAACGTCATGTTAGAGATCT
NF-*κ*B	F: GCTATAATCCTGGACTTCTG R: GAGGAAGGCTGTGAACATGA
Lc3	F: GAGTGGAAGATGTCCGGCTC R: CCAGGAGGAAGAAGGCTTGG
p62	F:GCTGCTCTCTTCAGGCTTACAG R: CCTGCTTCACAGTAGACGAAAG

**Table 3:** The effect of simvastatin on static histomorphometric parameters in rats.

Group	BV/TV(%)	Tb.Th (*μ*m)	Tb.N (mm^−1^)	Tb.Sp (*μ*m)
NC	41.88 ± 9.54	34.11 ± 7.89	12.54 ± 2.62	48.95 ± 15.09
HC	19.03 ± 8.62^*∗∗∗*^	26.36 ± 7.46^*∗*^	7.00 ± 1.86^*∗∗∗*^	130.44 ± 61.62^*∗∗∗*^
HC+SIM	43.98 ± 15.76^###^	37.58 ± 20.27^##^	12.89 ± 4.32^###^	48.50 ± 20.32^###^

Results are expression as mean ± SD of each group of rats (*n* = 10 per group). ^*∗*^*P* < 0.05; ^*∗∗*^*P* < 0.01; ^*∗∗∗*^*P* < 0.001 vs. the NC group. ^#^*P* < 0.05; ^##^*P* < 0.01; ^###^*P* < 0.001 vs. the HC group.

**Table 4:** Autophagy protein expression.

group	LC3II	p62
NC	0.011 ± 0.001	0.057 ± 0.031
HC	0.087 ± 0.060^*∗*^	0.092 ± 0.030^*∗*^
HC + SIM	0.067 ± 0.062	0.045 ± 0.032^###^

Results are expressed as mean ± SD of each group. ^*∗*^*P* < 0.05; ^*∗*^*P* < 0.01; ^*∗∗∗*^*P* < 0.001 vs. the NC group. ^#^*P* < 0.05; ^##^*P* < 0.01; ^###^*P* < 0.001 vs. the HC group.
